# Evaluation of whole-genome sequence data analysis approaches for short- and long-read sequencing of *Mycobacterium tuberculosis*

**DOI:** 10.1099/mgen.0.000695

**Published:** 2021-11-26

**Authors:** Nilay Peker, Leonard Schuele, Nienke Kok, Miguel Terrazos, Stefan M. Neuenschwander, Jessica de Beer, Onno Akkerman, Silke Peter, Alban Ramette, Matthias Merker, Stefan Niemann, Natacha Couto, Bhanu Sinha, John WA Rossen

**Affiliations:** ^1^​ University of Groningen, University Medical Center Groningen, Department of Medical Microbiology and Infection Prevention, Groningen, The Netherlands; ^2^​ University of Bern, Institute for Infectious Diseases, Bern, Switzerland; ^3^​ University of Groningen, University Medical Center Groningen, Department of Pulmonary diseases and Tuberculosis, Groningen, The Netherlands; ^4^​ University of Groningen, University Medical Center Groningen, TB Center Beatrixoord, Haren, The Netherlands; ^5^​ University of Tübingen, Institute of Medical Microbiology and Hygiene, Tübingen, Germany; ^6^​ Molecular and Experimental Mycobacteriology, Research Center Borstel, Borstel, Germany; ^7^​ The Milner Centre for Evolution, Department of Biology and Biochemistry, University of Bath, Bath, UK; ^8^​ Department of Pathology, University of Utah School of Medicine, Salt Lake City, UT, USA; ^9^​ IDbyDNA Inc., San Carlos, CA, USA

**Keywords:** *Mycobacterium tuberculosis*, drug-resistance prediction, nanopore sequencing, *de novo *assembly, cgMLST, cgSNP typing

## Abstract

Whole-genome sequencing (WGS) of *Mycobacterium tuberculosis* (MTB) isolates can be used to get an accurate diagnosis, to guide clinical decision making, to control tuberculosis (TB) and for outbreak investigations. We evaluated the performance of long-read (LR) and/or short-read (SR) sequencing for anti-TB drug-resistance prediction using the TBProfiler and Mykrobe tools, the fraction of genome recovery, assembly accuracies and the robustness of two typing approaches based on core-genome SNP (cgSNP) typing and core-genome multi-locus sequence typing (cgMLST). Most of the discrepancies between phenotypic drug-susceptibility testing (DST) and drug-resistance prediction were observed for the first-line drugs rifampicin, isoniazid, pyrazinamide and ethambutol, mainly with LR sequence data. Resistance prediction to second-line drugs made by both TBProfiler and Mykrobe tools with SR- and LR-sequence data were in complete agreement with phenotypic DST except for one isolate. The SR assemblies were more accurate than the LR assemblies, having significantly (*P*<0.05) fewer indels and mismatches per 100 kbp. However, the hybrid and LR assemblies had slightly higher genome fractions. For LR assemblies, Canu followed by Racon, and Medaka polishing was the most accurate approach. The cgSNP approach, based on either reads or assemblies, was more robust than the cgMLST approach, especially for LR sequence data. In conclusion, anti-TB drug-resistance prediction, particularly with only LR sequence data, remains challenging, especially for first-line drugs. In addition, SR assemblies appear more accurate than LR ones, and reproducible phylogeny can be achieved using cgSNP approaches.

## Data Summary

1. Illumina and ONT sequencing read files for 24 isolates have been deposited in the NCBI SRA database, accessible through BioProject number PRJNA720906.
2. The supplementary data deposited in the Figshare repository can be accessed at the following link: https://doi.org/10.6084/m9.figshare.17075987.v1.

**Impact Statement**

Tuberculosis, caused by *Mycobacterium tuberculosis* complex, is one of the leading causes of death from infectious diseases worldwide. Rapid and accurate diagnosis is essential for timely implementation of appropriate therapy which also prevents transmission and emergence/spread of drug-resistant tuberculosis. The rapid development of sequencing technologies, subsequent automated bioinformatics analysis of data, and efforts on its standardization have already transformed TB diagnosis in clinical settings. Our study assessed the relevance of recent advances in WGS of MTB, provided by both Illumina short-read (SR) and Oxford Nanopore Technologies (ONT) long-read (LR) sequencing technologies, for anti-TB drug-resistance prediction and MTB typing. Overall, our study provides a comparison of the currently in use bioinformatics tools employed for both SR and LR sequencing of MTB, aiming to guide investigators to choose the appropriate tools for different clinical diagnostic applications.

## Introduction

Tuberculosis (TB), caused by *Mycobacterium tuberculosis* (MTB), is one of the top-ranking causes of death from infectious diseases worldwide, with an estimated 10 million new cases and 1.5 million deaths in 2018 [1]. Rapid and accurate diagnosis is necessary for timely and appropriate antimicrobial therapy. This also prevents transmission and emergence/spread of multidrug-resistant (MDR)/extensively drug-resistant (XDR) tuberculosis [2]. However, conventional culture-based drug-susceptibility testing (DST) is relatively slow and sometimes challenging, affecting accuracy and reproducibility for certain drugs such as pyrazinamide [3]. The rapid molecular tests recommended by the WHO only cover a limited number of drugs and target a small number of resistance mutations, making the susceptibility prediction unreliable due to the false-negative test results [4]. Next-generation sequencing (NGS) technologies have been shown to have a high potential to overcome many of the challenges associated with conventional DST and the limitations of the current molecular tests by providing detailed sequence information for specific gene regions or the whole genome [2]. Since the complete genome sequencing of the first MTB [5], whole-genome sequencing (WGS) has been applied to a wide range of clinical situations: diagnosis, treatment, outbreak investigation and surveillance to guide clinical decision making and TB disease control [6, 7]. Moreover, WGS has also been anticipated to perform same-day diagnosis and surveillance of TB using the rapidly developing long-read sequencing technology of Oxford Nanopore Technologies (ONT) [8, 9]. Within the framework of the WHO End TB Strategy, which aims for a 95 % reduction in deaths and a 90 % reduction in incidence by 2035, the use of NGS technologies has been proposed to implement a universal DST for all TB patients [2]. Currently, most new MDR-TB cases originate from transmission events rather than from the emergence of resistance due to failed treatment. Therefore, improved diagnosis and treatment options with new drug regimens should be prioritized to combat the MDR-TB pandemic [10, 11].

Despite decreasing costs to integrate sequencing technologies into routine workflows, many laboratories still lack the computational resources and specialized staff required for analysing and managing sequencing data. There are several open-source or commercially available bioinformatics pipelines automating TB sequencing data manipulation and analysis in a single step, such as TBProfiler [12, 13], Mykrobe [14, 15] and MTBSeq [16] that facilitate the anti-TB drug-resistance prediction and MTB lineage classification from sequencing reads. The Ridom SeqSphere+, Bionumerics and CLC Genomics Workbench software are widely used in genotyping and outbreak investigations [17–20]. A standardized and validated data analysis approach is of utmost importance for laboratories to adopt NGS in TB diagnostics, surveillance and research [2, 16]. Currently available software and bioinformatics pipelines for MTB WGS data analysis have already been benchmarked in previous studies and evaluated for epidemiological typing [21] and for their performances in predicting anti-TB drug resistance with an extensive data set of MTB genomes, mainly from Illumina short-read (SR) sequence data [12, 15, 22–27]. Depending on the drug targets, the tools have exhibited variable performances to predict anti-TB drug resistance. For instance, for resistance prediction to first-line treatment drugs; rifampicin, isoniazid, pyrazinamide and ethambutol, TBProfiler showed 95.9, 93.7, 87.6, 92.1 sensitivity and 98.2, 98.1, 96.7, 91.7 % specificity, respectively. This compares to 100, 95, 82, 99 sensitivity, and 99, 100, 99, 99 % specificity, respectively, for the same drugs using the Mykrobe tool [12, 15]. However, there are limited numbers of studies using long-read (LR) sequencing data for anti-TB drug resistance prediction [9, 28] and for epidemiological typing [9].

In this study, we assessed the performance of TBProfiler and Mykrobe, whose mutation databases are up to date, for the prediction of anti-TB drug resistance for LR (ONT) sequencing data compared to SR (Illumina) sequencing data from the same sample set. This sample set (*n*=24) included the highest number of different MTB isolates (*n*=22) long-read sequenced for the evaluation of both TBProfiler and Mykrobe tools [12–15], and of both core-genome single nucleotide polymorphism (cgSNP) typing and core genome multi-locus sequence typing (cgMLST). We compared the robustness of two typing approaches based on cgSNP typing and cgMLST of either reads or assembled contigs for SR- and LR-sequence data. We evaluated different SR, LR and hybrid *de novo* assemblers for MTB genome assemblies and the subsequent typing approaches in this context. Overall, we give an overview of the most used bioinformatics tools employed for both SR and LR sequence data for anti-TB drug resistance and phylogenomic analysis of MTB to guide investigators to choose the appropriate tools depending on their requirements/aims/settings.

## Methods

### Samples

A total of 24 samples, consisting of 22 different *M. tuberculosis* (MTB) isolates, collected by the German National Reference Center for Mycobacteria (Borstel, Germany) (*n*=15) and by the National Reference Laboratory for Tuberculosis of The National Institute for Public Health and the Environment of the Netherlands (RIVM, Bilthoven, The Netherlands) (*n*=9) were used in this study. In this sample set, two of the samples were duplicates (Table S1 available in the online version of this article; QC-8 and QC-10; QC-5 and QC-9), and one sample (Table S1; QC-7), a mixture of two isolates, was obtained by mixing QC-1 and QC-8 suspended in TE (Tris EDTA) buffer to create a mixed sample of equal amounts. All MTB isolates were phenotypically tested for drug susceptibility (phenotypic DST) and genotyped into lineages/sub-lineages (Table S1). The DNA was isolated from MTB isolates grown in Löwenstein–Jensen medium using the cetyltrimethylammonium bromide (CTAB) method (Table S2) and genotyping of the isolates was performed in the corresponding reference laboratories by WGS.

### Whole-genome sequencing

DNA of the MTB isolates obtained from the reference laboratories for WGS was first checked for concentration and quality. The purity of the DNA was analysed with the Nanodrop spectrophotometer (Thermo Fisher Scientific, Waltham, Massachusetts, United States of America), and the DNA of the isolates obtained from Borstel was followed by two sequential 1 : 1 and 2 : 1 (v:v) ratio of AMPure XP bead (Beckman Coulter, Woerden, The Netherlands) purification steps. The DNA concentrations of all samples were measured with the Qubit dsDNA BR assay (Thermo Fisher Scientific), and fragment size analysis was performed using the gDNA ScreenTape assay on an Agilent 4150 TapeStation (Agilent, Santa Clara, CA, United States of America).

SR-sequencing (Illumina) libraries were prepared with the Nextera DNA Flex Library Preparation kit (Illumina, San Diego, CA, United States of America) according to the manufacturer’s instructions and sequenced on an Illumina NextSeq 500 platform (Illumina) to generate 151 bp paired-end reads.

LR-sequencing (ONT) libraries were prepared with the Ligation-Sequencing kit SQK-LSK109 complemented with the Native Barcoding Expansion kit EXP-NBD104, and the PCR Barcoding kit SQK-PBK004 (ONT, Oxford, United Kingdom), depending on the concentration of the input DNA. The ONT library preparation kits were supplemented with the following reagents from New England Biolabs (Ipswich, Massachusetts, United States of America); NEBNext End repair/ dA-tailing Module (E7546), NEB Blunt/TA Ligase Master Mix (M0367), LongAmp Taq 2X Master Mix (M0287), NEBNext FFPE Repair Mix (M6630), NEBNext Quick Ligation Module (E6056) and Agencourt AMPure XP beads (Beckman Coulter, Woerden, The Netherlands). The libraries were prepared according to the manufacturer’s instructions with the following modifications: dA-tailing and end repair using E7546 (NEB) with two-step incubation times at 20 °C and 65 °C 20 min each for the SQK-LSK109 kit and 10 min each for the SQK-PBK004 kit. All of the incubation steps with AMPure XP beads were increased to 10 min throughout the protocols in both library preparation kits. DNA libraries (four to five samples in each library pool) were sequenced with FLO-MIN106, R9.4 flow cells on two different instruments: GridION X5 or MinION (ONT). The sequencing procedure was set to run for 48 h with the real-time fast base calling mode enabled with Guppy v3.0.3 or v3.2.6 for MinION, while the GridION data was base-called using Guppy v3.0.3.

### Data analysis

**Fig. 1.**
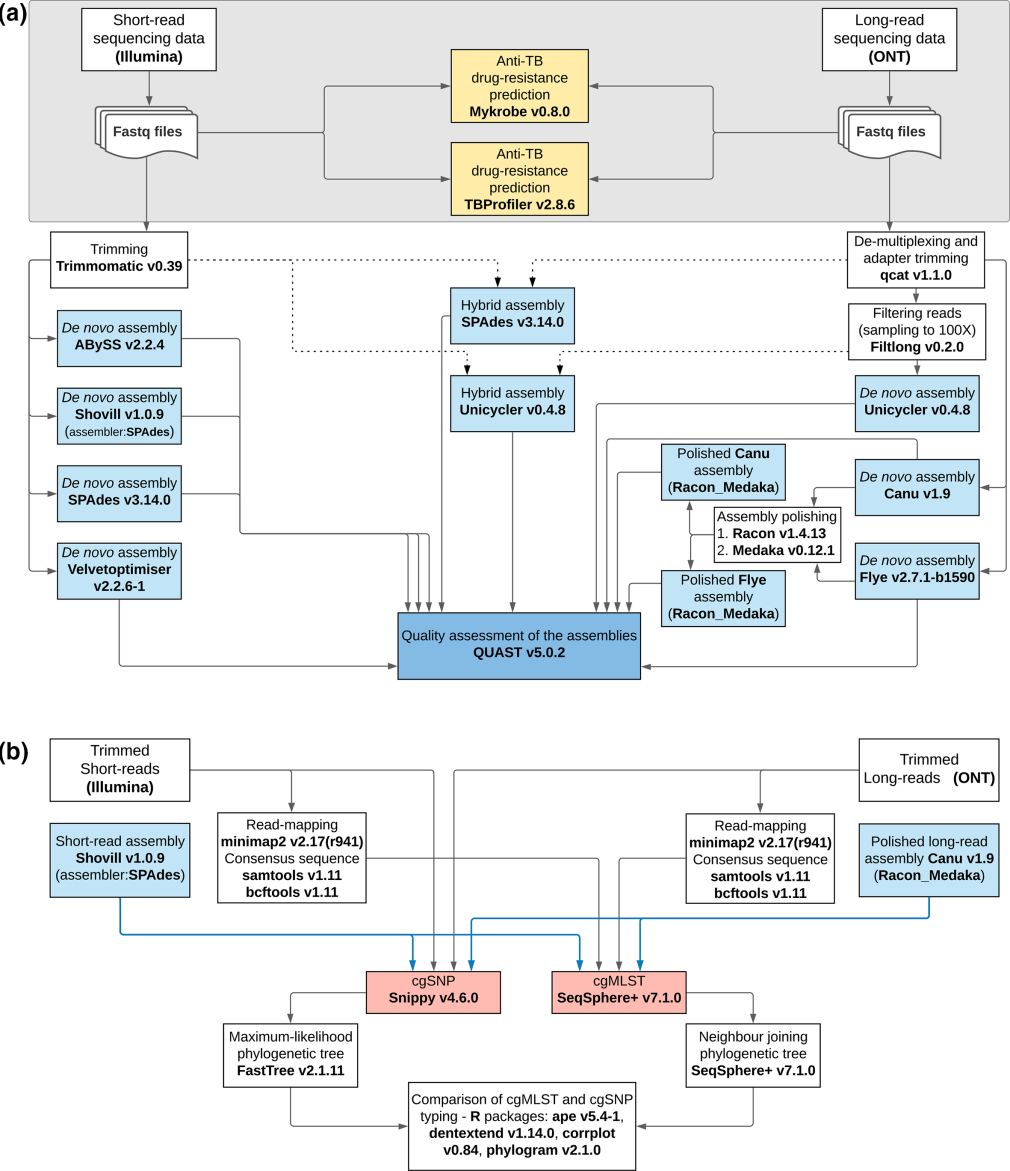
The workflow used for processing sequence data. (a) Tools in yellow are for detecting anti-TB drug-resistance mutations; tools in blue represent assemblers, and dashed lines indicate the hybrid assembly; (b) the tools in red are for typing and blue lines indicate the assembly-based typing.

The processing of the reads obtained by both SR and LR sequencing is illustrated in Fig. 1.

#### Pre-processing of the sequencing data

SR-sequencing raw reads were trimmed before *de novo* assembly using Trimmomatic v0.39 (http://www.usadellab.org/cms/index.php?page=trimmomatic) [29] (Table S3). The quality of the raw reads before and after trimming was checked with FastQC v0.11.9 (https://www.bioinformatics.babraham.ac.uk/projects/fastqc/). The raw reads were fed into the anti-TB drug-resistance prediction tools whose automated pipelines include pre-processing/trimming of the raw data (Table S3).

LR-sequencing raw reads were first demultiplexed into individual barcodes using qcat v1.1.0 https://github.com/nanoporetech/qcat), which also trimmed the barcode and adapter sequences (parameters: Table S3). The quality of the demultiplexed data was checked using NanoPlot v1.28.2 (https://github.com/wdecoster/NanoPlot) [30]. The mean read length was 2197 bp and 1002 bp for samples whose libraries were prepared with the SQK-LSK109 Ligation-Sequencing kit and the SQK-PBK004 PCR Barcoding kit, respectively. The average sequencing depth was 262× (maximum 1321× and minimum 27×, Table S4). The demultiplexed reads were used for anti-TB drug-resistance prediction and *de novo* assembly.

#### Detection of anti-TB drug-resistance mutations

The Mykrobe v0.8.0 (https://github.com/Mykrobe-tools/mykrobe) [14, 15] and the TBProfiler v2.8.6 (https://github.com/jodyphelan/TBProfiler) [12, 13] tools were evaluated for the detection of mutations conferring anti-TB drug resistance. Both tools, consisting of an automated pipeline for detecting SNPs associated with resistance, were run on the command-line interface using the default parameters (Table S3). The resistance prediction made by TBProfiler is based on mapping, where reads are aligned to the H37Rv reference using bowtie2, BWA or minimap2 (for ONT reads) and then variants are called using bcftools [12, 13]. Mykrobe does kmer-based resistance prediction using a list of variant sites presented as a set of sequence probes of length 2 k-1 [14, 15].

#### *De novo* assembly of the MTB genomes

Trimmed SRs were assembled using four different *de novo* assembly tools: ABySS v2.2.4 (https://github.com/bcgsc/abyss) [31], Shovill v1.0.9 [default: SPAdes] (https://github.com/tseemann/shovill), SPAdes v3.14.0 (https://github.com/ablab/spades) [32], and Velvetoptimiser v2.2.6–1 (https://github.com/tseemann/VelvetOptimiser) for Velvet assembler [33] with default parameters (Table S3). The SPAdes tool was also used for hybrid assembly with both SRs and LRs [34].

De-multiplexed and adapter trimmed LRs were *de novo* assembled using Unicycler v0.4.8 (https://github.com/rrwick/Unicycler) [35], Canu v1.9 (https://github.com/marbl/canu) [36] and Flye v2.7.1 (https://github.com/fenderglass/Flye) [37, 38]. For Unicycler assembly (LR and hybrid), samples with >100x read coverage were first randomly subsampled to 100x coverage using Filtlong v0.4.8 (https://github.com/rrwick/Filtlong) to reduce the computational burden of assembly. The contigs generated with Flye and Canu were polished with one round of Racon v1.4.13 (https://github.com/lbcb-sci/racon), followed by one round of Medaka v0.12.1 (https://github.com/nanoporetech/medaka) (Table S3).

#### Evaluation of *de novo* assembly tools

Eleven different *de novo* genome assemblies (four SR; five LR; two hybrid) for each of the 24 samples were evaluated using quast v5.0.2 (http://quast.sourceforge.net/quast.html) [39]. Three quality measures were assessed: completeness of the genome or genome fraction (%), number of mismatches per 100 kbp, and number of insertions/deletions (indels) per 100 kbp using the MTB strain H37Rv as reference (GenBank accession number NC_00962.3). The tables/figures and the statistical analysis were generated using GraphPad Prism v9 (GraphPad Software, San Diego, CA, USA). A *P*-value of ≤0.05 was considered significant.

#### MTB typing

The different assemblies were further evaluated for MTB typing using a gene-by-gene allele calling approach on Ridom SeqSphere +v7.1.0 software (Ridom GmbH, Münster, Germany). All of the genome assemblies (SR, LR and hybrid) for each of the samples were blastn aligned to an MTB cgMLST scheme (version 2.1) encompassing 2891 core genes [18]. The following quality thresholds were defined for a valid allele calling: (i) if the sequence length did not match the reference sequence length plus or minus three triplets, or if there were any ambiguous base supported by less than 60 % of the reads or if there were frameshifts, the target was reported as failed; (ii) if a genome assembly had more than 10 % cgMLST genes that did not match the quality criteria for allele calling, the genome was excluded. However, since most of the LR assemblies would have been excluded according to this last criterion, we kept all the genome assemblies, including those not exceeding the 90 % quality threshold. Besides, pairwise missing values were classified into their own category. The comparison tables of 11 different genome assemblies for the 24 samples were created, and cluster analysis was performed. The assemblies were compared based on the average number of good targets for allele calling, the average number of failed targets, and the average number of not found targets.

For the cgSNP typing, only the best SR and LR assemblies were chosen for comparison purposes. The best assembly was selected based on the highest average number of good targets for allele calling in the cgMLST analysis and based on the lowest average number of mismatches and indels per 100 kbp. Accordingly, Shovill and Canu+Racon+Medaka assemblies were fed to Snippy v4.6.0 (https://github.com/tseemann/snippy) for variant calling using the MTB strain H37Rv as reference (using the default parameters: a minimum mapping quality of 60; nucleotide quality with an error probability of ~5 %; a minimum of ten reads covering a site to be called; a minimum variant call quality of 100; genotypes 1/1). By default, Snippy ignores insertions/deletions variants. A whole-genome multi-FASTA alignment generated with Snippy was further processed by masking the repetitive regions of the MTB genome to exclude SNPs, which cause false positives, in those regions. Then, a maximum-likelihood (ML) phylogenetic tree was built using FastTree v2.1.11 with the GTR+CAT model (http://www.microbesonline.org/fasttree/) (Table S3).

Additionally, we used a read-mapping approach for the cgMLST and cgSNP typing. For the cgMLST analysis, LRs and SRs were mapped to the H37Rv genome using minimap2 v2.17 (https://github.com/lh3/minimap2) [40]. Then the variants were called, and the consensus sequences were extracted using samtools v1.11 (https://github.com/samtools/samtools) and bcftools v1.11 (https://github.com/samtools/bcftools) [41] (Table S3). The consensus sequences were processed on Ridom SeqSphere +v7.1.0 software for gene-by-gene allele calling, and neighbour joining (NJ) trees were drawn. For the cgSNP analysis, trimmed SR and LR were processed using Snippy v4.6.0, as explained above.

The ML-trees of the cgSNP analysis and the NJ-trees of the cgMLST obtained from either reads or assembled contigs for both SR and LR sequence data were compared using R packages: ape v5.4–1, dendextend v1.14.0, corrplot v0.84, and phylogram v2.1.0 [42–45]. The concordance between trees as means of distance matrices was evaluated on dendrogram plots and visualized using tanglegrams (dendextend v1.14.0). The symmetric difference known as the Robinson–Foulds distance between the dendrograms was computed (Table S12) using the dist.dendlist function (dendextend v1.14.0). Then, a correlation matrix was created by calculating the cophenetic correlation coefficient, a measure that tells how well the pairwise distances on a dendrogram match the original distances used to construct it [46], using the cor.dendlist function (dendextend v1.14.0) for all trees and visualized using corrplot v0.84. The values range from 1, perfect positive correlation, to −1, negative perfect correlation with near 0 values meaning that the two trees are not statistically similar [43]. The correlation of the distances between samples in one matrix to the other was also computed with the Mantel test using the python qiime package called ‘compare_distance_matrices.py’ v1.9.1 (Tables S3 and S13).

## Results

### Anti-TB drug-resistance prediction for LR compared to SR sequence data

**Fig. 2.**
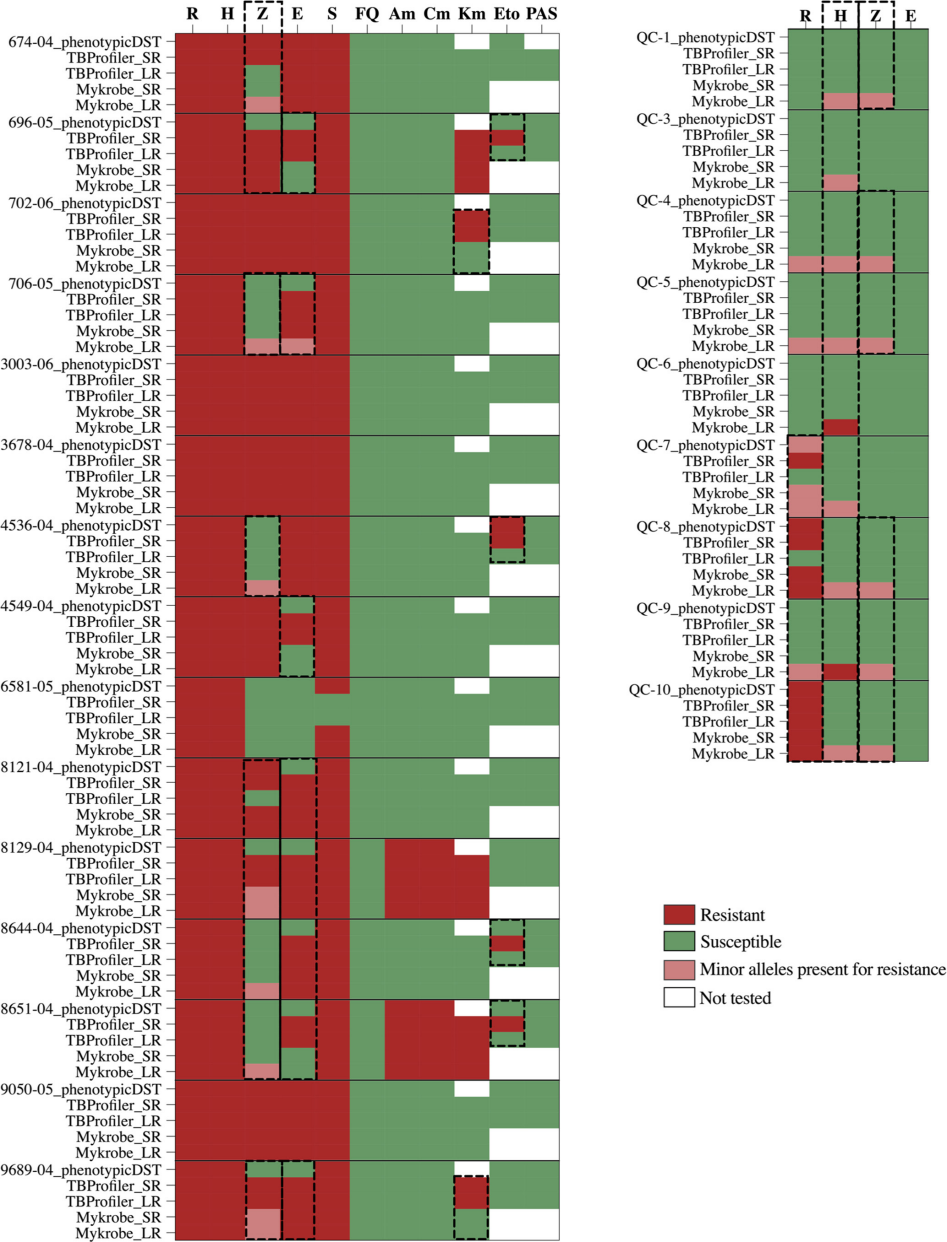
Evaluation of anti-TB drug-resistance profiles predicted by TBProfiler and Mykrobe tools from both short-read (SR) and long-read (LR) sequence data compared to the corresponding reference laboratory phenotypic DST results. Drugs tested for antibiotic resistance are as follows: R, rifampicin; H, isoniazid; E, ethambutol; Z, pyrazinamide; S, streptomycin; FQ, fluoroquinolones (specified as moxifloxacin, ofloxacin, ciprofloxacin in the Mykrobe tool); Am, amikacin; Cm, capreomycin; Km, kanamycin; Eto, ethionamide; PAS, p-aminosalicylic acid. The discrepancies in resistance prediction for each drug were highlighted with dashed lines.

The comparative analysis of the two most recently updated anti-TB drug-resistance prediction tools, TBProfiler and Mykrobe, for the 24 samples, including their phenotypic drug-susceptibility profiles, is presented in Fig. 2. We looked at the SNP calls made using LR or SR sequence data and their concordance to phenotypic DST (Fig. 2, Table 1).

**Table 1.** The number of isolates for drug-resistance prediction concordant/disagreement with the phenotypic DST for the drugs present in the drug panels of both tools

Table 1a: The number of isolates (%) for drug-resistance prediction concordant/disagreement with the phenotypic DST for the first-line drugs.												
	Rifampicin			Isoniazid			Pyrazinamide			Ethambutol		
	Concor. (%)	Disagre. (%)	Other*	Concor. (%)	Disagre. (%)	Other*	Concor. (%)	Disagre. (%)	Other*	Concor. (%)	Disagre. (%)	Other*
**TBProfiler** Short-read	23/24 (96.0)	1/24 (4.0)	na	24/24 (100.0)	0	na	21/24(87.5)	3/24(12.5)	na	16/24 (67.0)	8/24(33.0)	na
**TBProfiler** Long-read	22/24 (92.0)	2/24 (8.0)	na	24/24 (100.0)	0	na	19/24 (79)	5/24(21.0)	na	16/24 (67.0)	8/24(33.0)	na
**Mykrobe** Short-read	24/24 (100.0)	0	0	24/24 (100.0)	0	0	20/22 (91)	2/22(9.0)	2	19/24 (79.0)	5/24(21.0)	0
**Mykrobe** Long-read	21/21 (100.0)	0	3	15/17 (88.0)	2/17(12.0)	7	10/11 (91)	1/11(9.0)	13	19/23 (83.0)	4/23(17.0)	1
**Table 1b: The number of isolates (%) for drug-resistance prediction concordant/disagreement with the phenotypic DST for the second-line drugs.**												
	**Streptomycin**			**Fluoroquinolones**			**Amikacin**			**Capreomycin**		
	**Concor. (%)**	**Disagre. (%)**	Other*	**Concor. (%)**	**Disagre. (%)**	Other*	**Concor. (%)**	**Disagre. (%)**	Other*	**Concor. (%)**	**Disagre. (%)**	Other*
**TBProfiler** Short-read	14/15 (93.0)	1/15 (7.0)	na	15/15 (100.0)	0	na	15/15 (100.0)	0	na	15/15 (100.0)	0	na
**TBProfiler** Long-read	14/15 (93.0)	1/15(7.0)	na	15/15 (100.0)	0	na	15/15 (100.0)	0	na	15/15 (100.0)	0	na
**Mykrobe** Short-read	15/15 (100.0)	0	0	15/15 (100.0)	0	0	15/15 (100.0)	0	0	15/15 (100.0)	0	0
**Mykrobe** Long-read	15/15 (100.0)	0	0	15/15 (100.0)	0	0	15/15 (100.0)	0	0	15/15 (100.0)	0	0

∗Minor allele calls made for drug resistance in phenotypically susceptible or resistant isolates

Concor, Concordance; Disagre, Disagreement; na, Not applicable.

Most of the discrepancies between phenotypic DST and drug-resistance prediction were observed for first-line drugs, and mainly when using LRs (Fig. 2, Table 1a). The resistance prediction made using SRs was 100 % concordant with the phenotypic DST for isoniazid using both tools (Fig. 2) and 96 and 100% concordant for rifampicin using TBProfiler and Mykrobe tools, respectively. The resistance prediction made using LRs was 92 % (22/24) and 100 % (21/21) concordant for rifampicin and 100 % (24/24) and 88 % (15/17) concordant for isoniazid with the phenotypic DST using TBProfiler and Mykrobe, respectively. TBProfiler did not detect the rifampicin resistance mutations in two samples (QC-7,8). On the other hand, Mykrobe identified minor allele calls for rifampicin resistance in three (QC-4,5,9) phenotypically susceptible isolates (Fig. 2). Mykrobe classified the presence of minor alleles (having frequencies around 8–10 % with coverages around 30–50× [15]) conferring drug resistance in a separate category referring to minor allele-driven resistance (Table 1), while TBProfiler classified the mutations as ‘R’ regardless of the allele frequency. A good example are strains 8129–04 and 9689–04, where Mykrobe predicted the pyrazinamide pattern as ‘r’ while TBProfiler as ‘R’ (although the frequency of the allele is minor, see Table S5). Furthermore, ethambutol resistance was predicted in four (8121–04, 8129–04, 8644–04, 9689–04) by both tools using SR and LR, although they were phenotypically susceptible (Fig. 2). Three ethambutol susceptible strains (696–05, 4549–04, 8651–04) by DST had predicted resistant mutations (*embB*_p.Asp328Gly and *embB*_p.His1002Arg) by TBProfiler that are not present in the Mykrobe database. The minor allele-driven resistance calls were mainly made on LR sequence data using Myrkobe with a rate as high as 29 %(7/24) and 54 %(13/24) for isoniazid and pyrazinamide, respectively (Table 1a; other) and were excluded from evaluation of concordance/disagreement to phenotypic DST. TBProfiler also reported variants not associated with known drug resistance (data not shown). Nevertheless, the minor allele calls for rifampicin resistance in sample QC-7 predicted by Mykrobe agreed with the test result of the reference laboratory, where the DNA of rifampicin resistance isolate QC-1 and rifampicin susceptible isolate QC-8 were mixed almost in equal amounts to obtain sample QC-7 (Table S1). The drug-resistance prediction was discordant for rifampicin resistance in duplicates QC-8 and QC-10 and for isoniazid resistance in duplicates QC-5 and QC-9 using TBProfiler and Mykrobe on LR data, respectively.

Resistance prediction to second-line anti-TB drugs was more consistent, with resistance prediction to streptomycin, fluoroquinolones, amikacin, capreomycin and kanamycin made by both tools in complete agreement with the phenotypic DST except for one isolate missed by TBProfiler for streptomycin resistance (Fig. 2, Table 1b) and two isolates with predicted kanamycin resistance by TBProfiler, but missed by Mykrobe (Fig. 2). These differences are explained by database differences: the mutation associated with streptomycin resistance identified by Mykrobe (*gid*_p.D85A, Table S5) is not present in the TBProfiler database, while the two mutations associated with kanamycin resistance identified by TBProfiler (*eis*_−12C>T and *eis*_−37G>T, Table S5) are not present in the Mykrobe database.

Both tools provided lineage information of the isolates, and the sub-lineages and spoligotypes were further provided by TBProfiler. The average CPU time of the analysis using TBProfiler and Mykrobe was similar for SR data, whereas Mykrobe was five times faster than TBProfiler for LR data (Table S6).

### Quality assessment of SR, LR, and hybrid assemblers for *de novo* assembly of MTB genomes

Eleven assemblies were generated for each sample: four with SR using ABySS, Shovill, SPAdes and Velvet (through VelvetOptimizer); five with LR using Canu, Flye, Unicycler and two additional polished versions of the Canu and Flye assemblies corrected with one round of Racon followed by one round of Medaka; and two hybrid assemblies with both SR and LR sequence data using Unicycler and SPAdes. All assemblies were evaluated compared to the genome of the reference MTB strain H37Rv using the quality-assessment tool quast (Table S7). The differences in the performance of the assembly tools were further evaluated by performing a Kruskal–Wallis test followed by a Dunn’s multiple comparisons test (α=0.05) (Tables S8–S10).

**Fig. 3.**
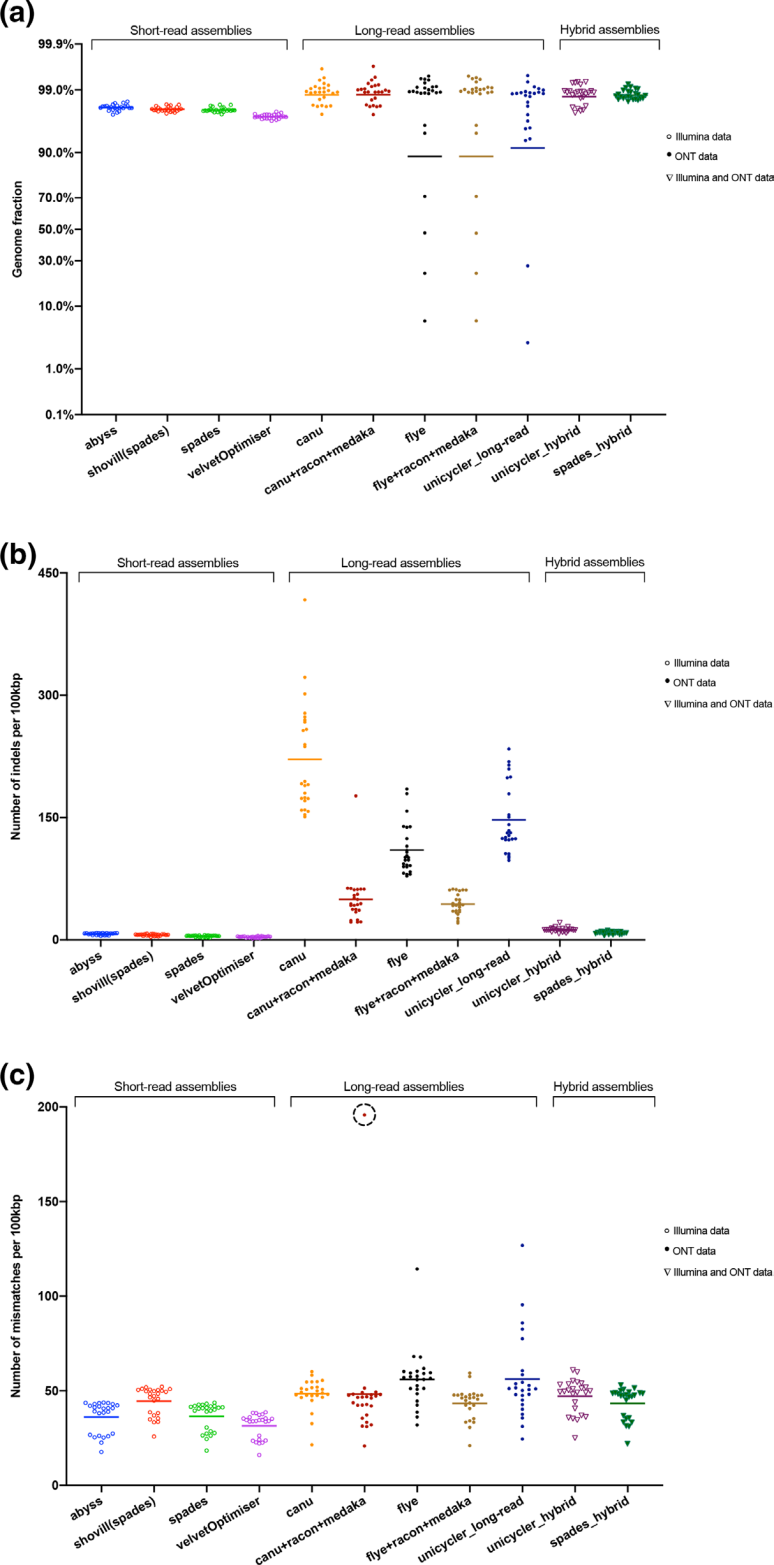
Quality assessment of the assemblers: (a) the genome fraction; (b) the average number of indels per 100 000 aligned bases; (c) the average number of mismatches per 100 000 aligned bases according to the assembly contigs mapping to the reference MTB strain H37Rv. The sample exhibiting a higher number of mismatches after correction of the Canu assembly is indicated with a dashed circle (QC-7).

The SR assemblies covered, on average, 97–98 % of the genome (Fig. 3a) and did not significantly differ from each other (*P*>0.05). The Canu assemblies were the most accurate in terms of average genome fraction (98.8%), while the assembly correction with Racon and Medaka did not improve the genome fraction. Nevertheless, the genome fraction provided by the polished Canu assemblies differed significantly from the SR assemblies (Table S8). Surprisingly, four and two assemblies had less than 70 % genome fractions when using Flye (including the polished version) and Unicycler for LR, respectively (Fig. 3a). The low QC of these samples probably caused this. In terms of genome fraction, the hybrid assemblies were not significantly different from the LR assemblies (Table S8).

The number of indels per 100 kbp was lowest in the SR assemblies, while it varied in the LR assemblies, for which the Canu assemblies contained the highest average number of indels per 100 kbp (Fig. 3b). For the Canu and Flye assemblies, the average number of indels per 100 kbp decreased significantly after polishing with Racon, followed by Medaka (Fig. 3b, Table S9).

The SR assemblies, in general, had the lowest number of mismatches per 100 kbp, which was lowest with Velvet. The average number of mismatches per 100 kbp significantly decreased in the Flye assemblies after polishing, whereas the change was trivial in the Canu assemblies (Table S10) except for sample QC-7 (Table S7). In this sample, the polishing of the Canu assembly increased the number of mismatches per 100 kbp (Fig. 3c), since this sample consisted of a mixture of two MTB isolates. This sample was included in further analysis to mimic what would happen in ordinary circumstances if no information about the sample would have been provided.

The hybrid assemblies did not differ significantly from each other, but SPAdes assemblies, in general, were slightly better than Unicycler assemblies concerning the number of mismatches and indels per 100 kbp (Fig. 3, Table S9). In contrast, the number of contigs was lower with Unicycler (Table S7). In terms of analysis time, Velvet and SPAdes as SR assemblers, Canu as LR assembler, and Unicycler as hybrid assembler were the slowest in assembling genomes (Tables S6).

### MTB typing

#### Evaluation of *de novo* assembly tools for MTB typing

**Fig. 4.**
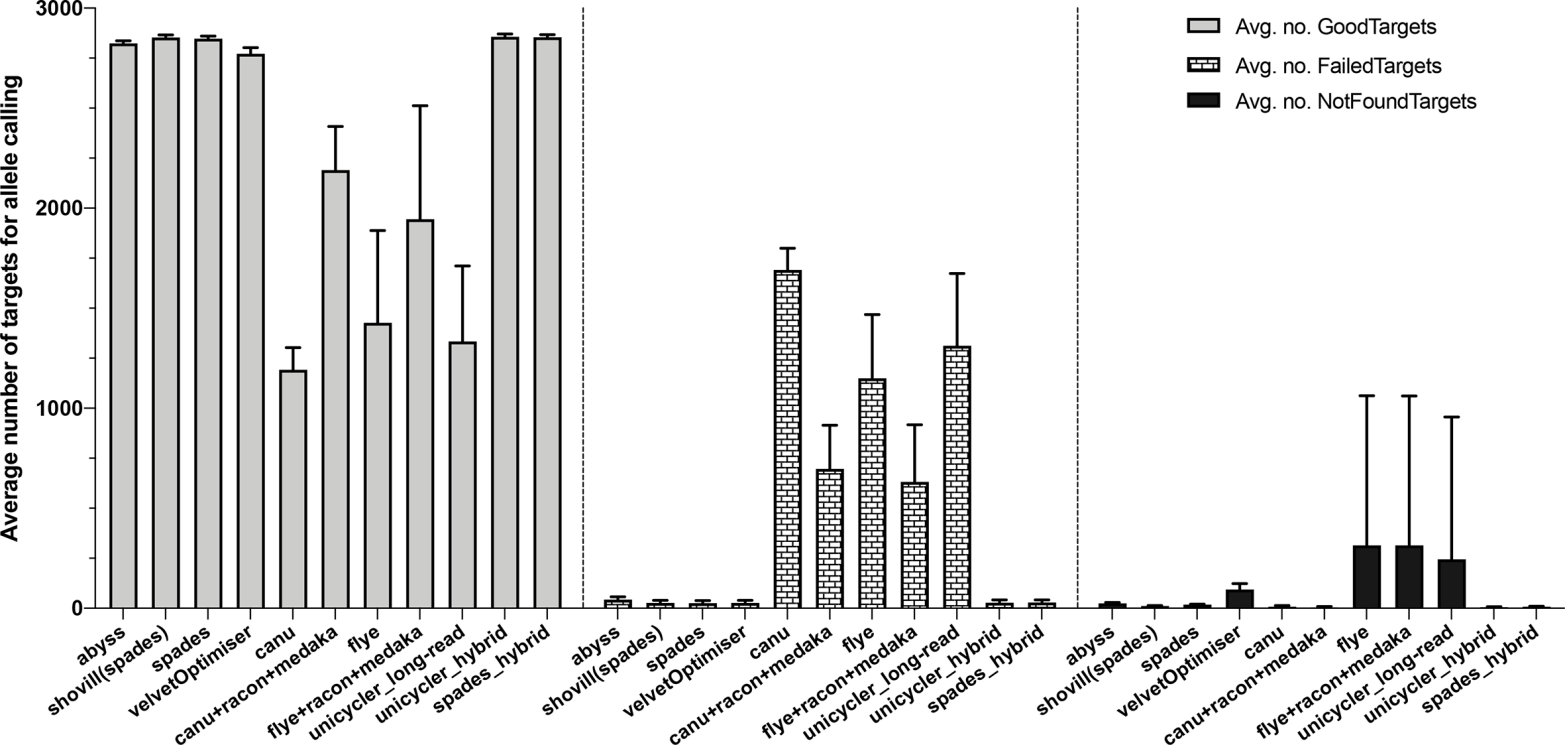
The average number of ‘Good’ (targets that passed the quality criteria), ‘Failed’ (if the target match does not meet at least one of the requirements for the quality thresholds defined for a valid allele calling), and ‘Not Found’ (if the target match does not reach the quality thresholds or the target is not present at all) targets for allele calling for cgMLST analysis of 24 samples using different assemblers on Ridom SeqSphere+.

All the MTB genome assemblies (SR, LR and hybrid) were evaluated using a gene-by-gene allele calling approach with a cgMLST scheme composed of 2891 gene targets (Table S11). The average number of gene targets valid for allele calling differed significantly (*P*≤0.05) between the SR and LR assemblies (Table S11). In LR assemblies, there were a significant number of targets that either ‘Failed’, i.e. the target did not meet at least one of the requirements for the quality thresholds defined for allele calling, or was ‘Not Found’, i.e. either the target did not match one of the quality thresholds, or the target was not present at all (Fig. 4). The allele calling with hybrid assemblies was comparable to the one with SR assemblies. There were, however, slight differences in the alleles called (Table S11).

**Fig. 5.**
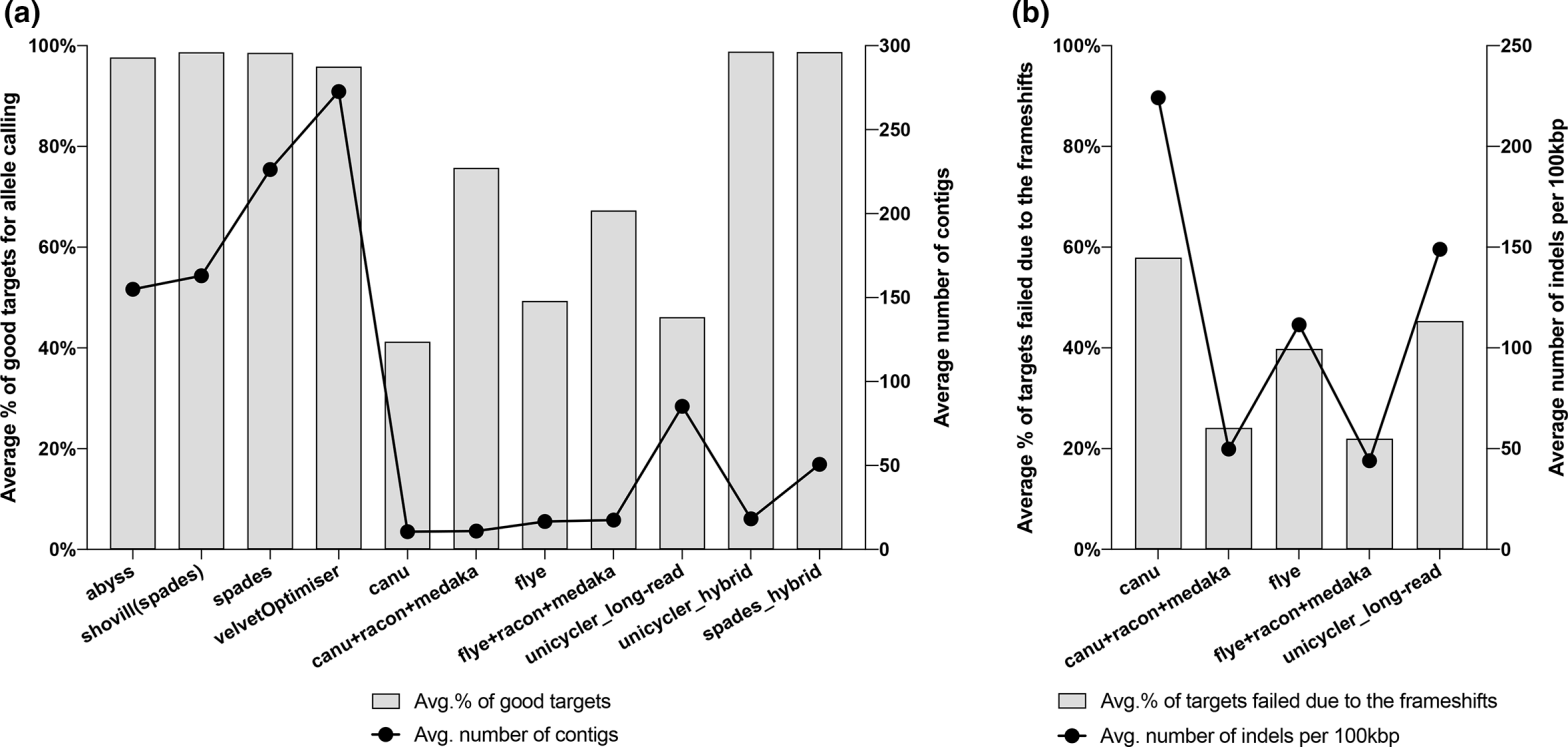
The average number of contigs generated by different assemblers and the average proportion of targets passing the quality criteria (good targets) for allele calling on Ridom SeqSphere+ (a). The association between the average proportion of failed targets due to frameshifts for allele calling and the average number of indels per 100 kbp generated with different LR assemblers (b).

The proportion of gene targets that passed the quality criteria for allele calling was as low as 41 % in LR assemblies, particularly with Canu, which was increased to 76 % after correction with Racon and Medaka (Fig. 5a). We did not observe an association between the average number of contigs generated by different assemblers and the percentage of good targets for allele calling in the assemblies (Fig. 5a). However, 22–58 % of the targets failed for allele calling due to frameshifts related to the higher number of indels per 100 kbp in the LR assemblies (Fig. 5b).

#### Evaluation of MTB cgMLST and cgSNP typing

For phylogenomic comparison, we considered two assemblers: Shovill for SR and Canu with Racon and Medaka polishing for LR, as they outperformed the other tools in terms of genome completeness, the lowest number of mismatches and indels per 100 kbp, and the highest number of gene targets valid for allele calling in cgMLST analysis.

**Fig. 6.**
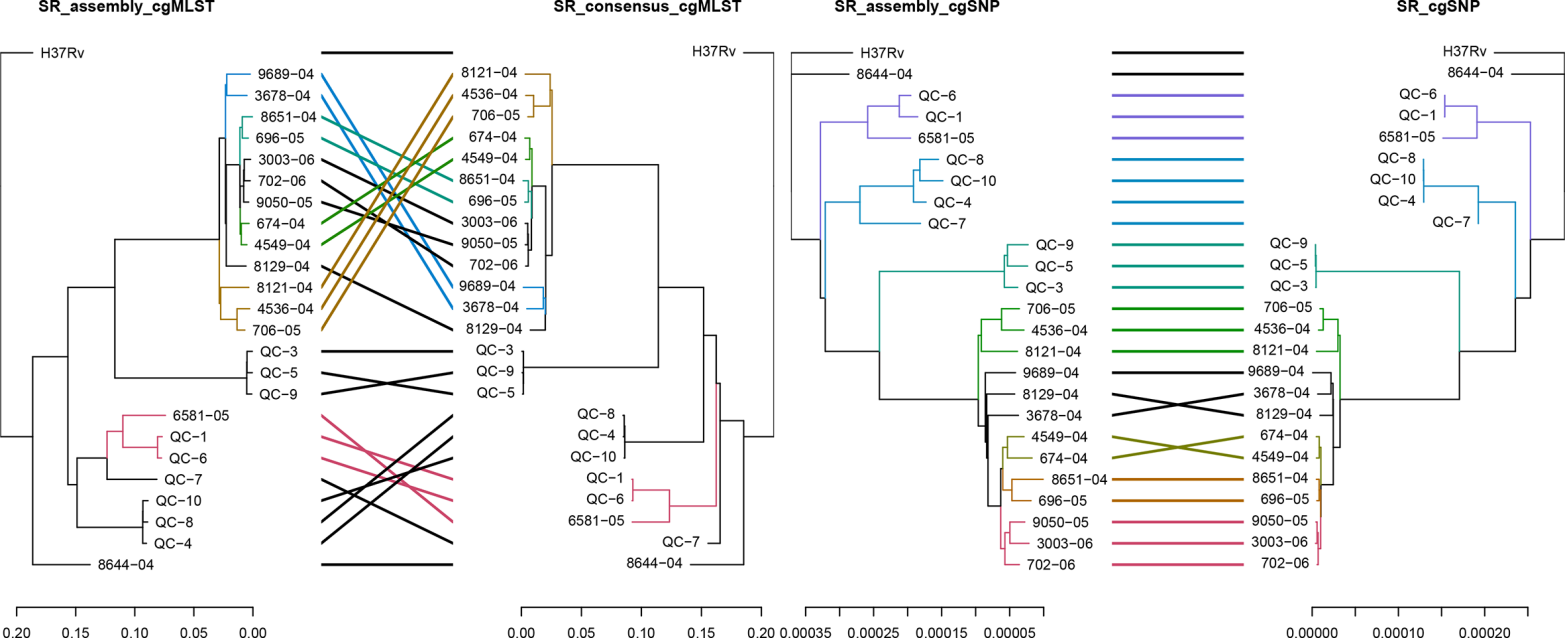
Comparison of the NJ trees of the isolates obtained with SeqSphere +cgMSLT analysis (left) and the ML trees obtained with cgSNP analysis (right) using reference-based mapping of SRs or SR assemblies (assembler: Shovill). The branches of both trees and connecting lines are coloured based on the common subtrees.

**Fig. 7.**
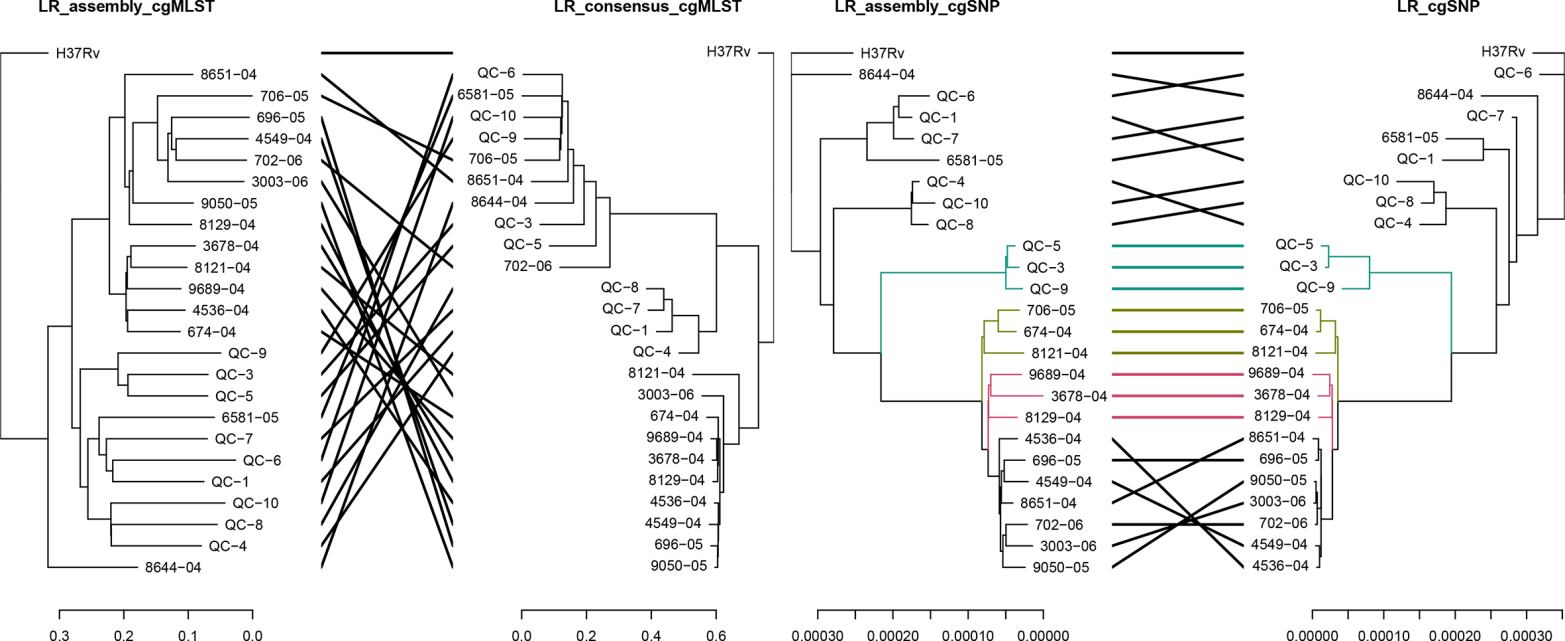
Comparison of the NJ trees of the isolates obtained with SeqSphere +cgMSLT analysis (left) and the ML trees of the isolates obtained with cgSNP analysis (right) using reference-based mapping of LRs or LR assemblies (assembler: Canu corrected with Racon and Medaka). The branches of both trees and connecting lines are coloured based on the common subtrees.

The comparison of cgMLST and cgSNP based phylogenies of the 24 samples, generated by reference-based mapping or assembly, can be seen in Figs. 6–7. The clustering of the isolates between assembly- and read mapping-based approaches was more consistent in the cgSNP trees than in the cgMLST trees for both SR and LR data. The read mapping-based approach, mainly for cgMLST analysis using LRs, resulted in erroneous clustering. The clustering of the isolates was more concordant with the lineages (Table S1) in the assembly-based approach than the read-based approach, despite the high genetic distances (Fig. 7). The Robinson–Foulds distances referring to the symmetric differences between trees revealed the highest difference between cgMLST trees of LR and the highest similarity between cgSNP trees of SR data (Table S12). Considering a minimum distance expected between the duplicate samples (Table S1: QC-5 and QC-9; QC-8 and QC-10), the cgSNP analysis by read mapping performed better among the typing approaches using SRs. However, the cgSNP analysis of the assemblies was the most precise approach for LRs considering the reference clustering information (Fig. 7, Table S1).

**Fig. 8.**
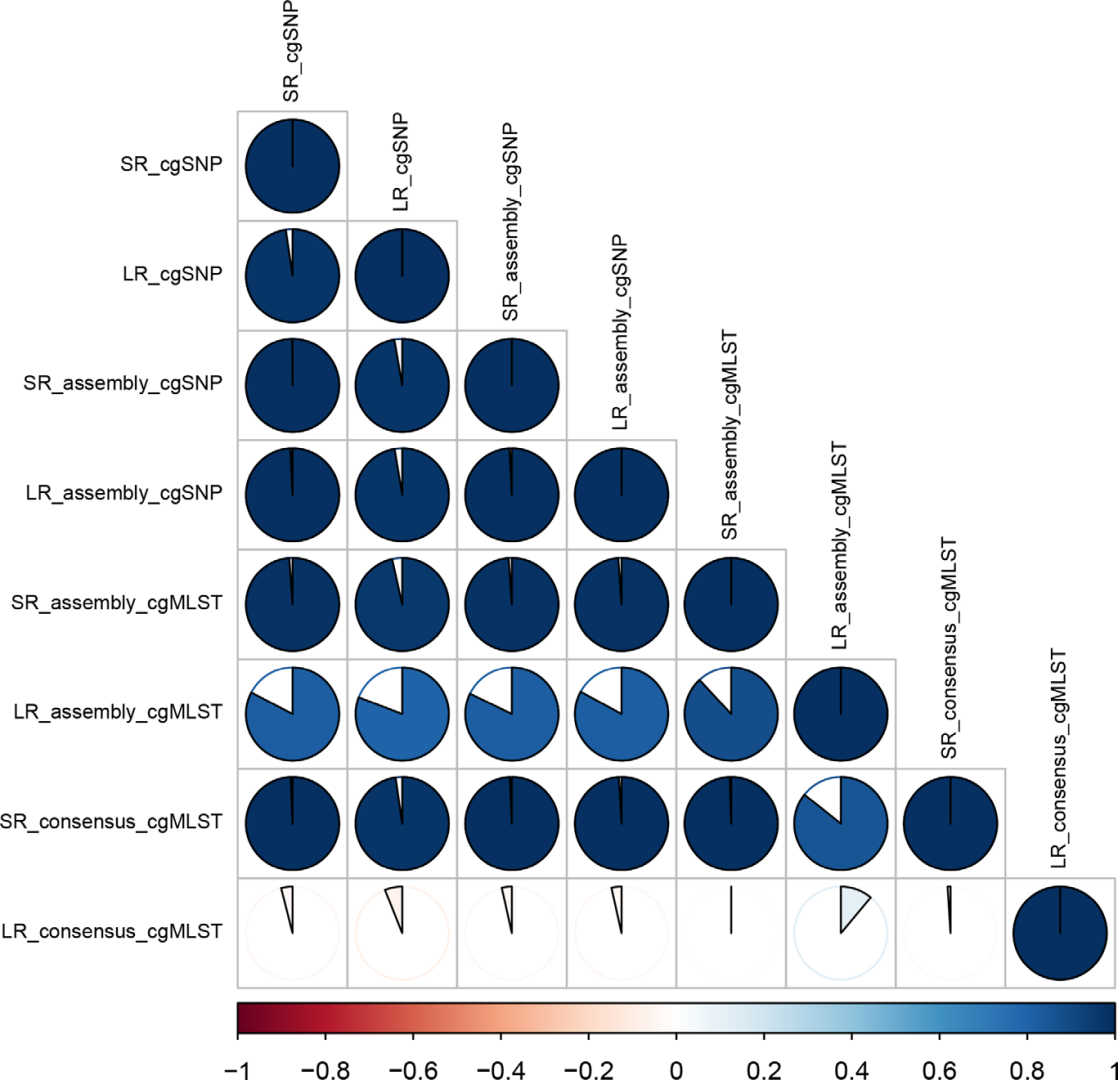
Correlation matrix of the trees visualized using corrplot v.084. The cophenetic correlation was calculated to create a correlation matrix (correlation coefficient: cophenetic) using the cor.dendlist function of dendextend v1.14.0 [SR_assembly: assembly of Shovill, LR_assembly: assembly of Canu (corrected with Racon and Medaka)]. Positive correlations are displayed on a blue scale, while negative correlations are displayed on a red scale. White means the trees are not statistically similar.

The similarity between the cgMLST and the cgSNP trees through clustering referring to the distances among isolates was given by the cophenetic correlation in Fig. 8. Accordingly, there was a perfect positive correlation in clustering between the trees of SRs, followed by the cgSNP trees of LRs and LR assemblies. The cgMLST trees of LRs and LR assemblies were significantly different from the other trees with a correlation measure of around −0.8 and 0.4, respectively (Fig. 8). The distance matrices of cgMLST and cgSNP typing of SRs and SR assemblies were significantly correlated (Mantel *r* statistics>0.97, *P*=0.001). The best correlation for LR data was observed between cgSNP typing of LR assemblies and typing of SRs (Mantel *r* statistics=0.97–0.99, *P*=0.001) (Tables S13 and Table S14).

## Discussion

Recent advances in NGS technologies have expanded the application of WGS as a diagnostic tool guiding TB treatment. This approach has already been implemented in the United Kingdom and the Netherlands through genotypic drug susceptibility testing to first-line drugs [47]. However, translation of the genetic information into drug-susceptibility phenotype requires sophisticated and robust bioinformatics pipelines [27] standardized and validated not only for Illumina-SR sequencing but also for ONT-LR considering its cost-effectiveness [9], portability in resource-limited settings, and ability to provide same-day diagnostics [8, 9, 12].

In this context, we first evaluated the Mykrobe and TBProfiler tools predicting anti-TB drug-resistance on a set of different isolates (*n*=22) sequenced with both short- and long-read technologies, which has not been performed before. Both tools had comparable performances. The drug-resistance prediction with SRs was more concordant with the phenotypic DST than the LRs. Regardless of the tool, the disagreements between phenotypic DST and resistance prediction were mainly observed for pyrazinamide and ethambutol. It is challenging to further evaluate the discrepancies for pyrazinamide as the WHO-recommended methodology for pyrazinamide phenotypic DST lacks reproducibility and is associated with a high rate of false-positive results attributed to the high inoculum concentration [2, 3]. The critical proportion at which the resistance is detected for anti-TB drugs is 0.01, whereas it is 0.1 for pyrazinamide. This higher proportion makes phenotypic DST less sensitive to low-frequency populations, which was considered while developing the Mykrobe tool that classifies minor-allele driven resistance separately [15]. The minor allele-driven resistance calls were mostly made on error-prone LRs with a rate as high as 54 % (13/24) for pyrazinamide (Table 1b; other). Besides the challenges of phenotypic susceptibility testing mentioned above, one should also consider the sequencing artefacts behind this high false-positive rate observed for predictions made on LRs as it was also observed previously [9]. A balance between sensitivity and specificity should be considered and for the time being a combination of both tools could be used for drug-susceptibility prediction. A recent study revealed another aspect of low accuracy of pyrazinamide resistance prediction using online tools (including TBProfiler) [48]. Manual inspection of the variant calling lists obtained by different tools showed that many drug-resistance-associated variants were missing in the default reports due to the automatic interpretation of drug resistance upon predefined mutation catalogues and was not due to the inability of the software algorithms to detect genetic variants [48]. Moreover, studies showed that the presence of individual or multiple mutations in the ethambutol resistance target *embB* gene and the *embCAB* operon could increase the ethambutol MIC, leading to different levels of ethambutol resistance [49, 50]. In our analysis, the high-frequency mutations detected by both TBProfiler and Mykrobe in ethambutol-susceptible isolates were likely to cause minor increases in MIC, but not enough to develop a complete resistance phenotype. Both TBProfiler and Mykrobe provide a plain text analysis summary report, which can be understandable for clinicians to guide the treatment regimen. It is essential to generate an easily interpretable report in clinical settings where the bioinformatics expertise is usually missing. However, users should also be aware of the cons of an automated interpretation to avoid misinterpretations because of the reasons mentioned above. A final point to consider when implementing WGS data analysis tools in clinical microbiology is the time for analysis. Mykrobe presented the best performance with an average of 4 and 5 min of CPU time compared to an average of 7 and 21 min of CPU time per sample with TBProfiler to analyse SR and LR sequence data, respectively (Table S6).

In the following quality assessment of the assemblies, the SR assemblers ABySS, Shovill, SPAdes and Velvet presented overall comparable performances. Considering the assembly time (CPU time) spent by each tool (Table S6), ABySS and Shovill were frontrunners. Shovill has the advantage over Abyss of performing an assembly correction step using Pilon (https://github.com/tseemann/shovill). For the LRs, Canu assemblies followed by Racon and Medaka correction worked as the best approach for *de novo* assembly of the MTB genomes with higher genome fractions and a lower number of indels and mismatches per 100 kbp. Nevertheless, Canu was much slower (approx. 5 times) than Flye and Unicycler assemblers. However, it is important to highlight that none of the approaches was able to separate the two MTB genomes present in sample QC-7. The performance of different LR assemblers on diverse bacterial genomes with different GC content, including XDR-TB, was evaluated in previous studies [51, 52]. In addition to our current evaluation, a further analysis could be performed to explore the ability of LR assemblers to resolve and to annotate the repetitive genomic regions [52], which are one of the characteristics of MTB genomes.

Hybrid assemblies are often considered superior to either SR assemblies and LR assemblies in terms of genome completeness and accuracy [53]. That is what we observed in our analysis. Interestingly, however, minor differences in the alleles called by Ridom SeqSphere +were identified in the hybrid assemblies when compared to the SR assemblies. A more in-depth analysis showed that the hybrid assemblies had mismatches compared to the short-read assemblies that could not be attributed to low coverage since there was good sequencing depth of both SR and LRs on those genome positions. These were caused by an erroneous single nucleotide variant calling by the assemblers. For this reason, we performed a *k*-mer optimization on both hybrid assemblers (SPAdes and Unicycler), but it still did not improve the single nucleotide variant calling. This phenomenon has been observed before on a benchmarking study comparing hybrid assemblers [53]. There is currently no solution to this observation and it should be taken into account when (i) updating available hybrid assemblers or when developing new ones (ii) using different types of assemblies (i.e. short and hybrid assemblies) for comparing resulting phylogenies, as it will introduce errors that could lead to a misinterpretation of the results.

cgMLST and cgSNP typing using reads and assemblies (SR: Shovill; LR: Canu corrected with Racon and Medaka) were evaluated. Even though cgSNP typing produced comparable results for both SR and LR assemblies, the cgMLST of SR and LR assemblies differed significantly. A lower number of gene targets valid for allele calls were detected in a predefined set of genes for LR assemblies (on average 76%) compared to SR assemblies (on average 99%). Ultimately, the genetic distances between the isolates were higher in LR assembly NJ trees. Based on the clustering information provided on the isolates, we determined that the read-mapping approach, instead of the assembly approach, is the most appropriate way of performing cgMLST and cgSNP typing of MTB from SRs, as used by many laboratories [21]. The higher accuracy of typing by read-mapping than assembly could be attributed to incorrect nucleotide calling during assembly or error correction [53, 54]. When we further considered the minimum genetic distance expected between duplicate samples, the cgSNP typing by reference-based mapping of SRs would be the best option for MTB genome typing. We furthermore observed that cgSNP typing of LRs and LR assemblies had the highest correlation with the SR-based typing (Mantel *r* statistics=0.97–0.99, *P*=0.001) (Tables S13 and S14). Therefore, a cgSNP-based typing approach should be chosen for surveillance and transmission investigations using LR sequence data.

Short-read sequencing has a high GC bias but is highly accurate for single nucleotide variant calling and small indels. Long-read sequencing, instead, has the advantage of resolving structural variations and variants in repetitive regions. For example, the highly repetitive PE/PPE gene families, which comprise approximately 10 % of the coding regions in *M. tuberculosis *, have been suggested to play a role in virulence [55] and their association with drug resistance remains largely unexplored. These regions are poorly resolved by short-reads and are often excluded by bioinformatics studies of *M. tuberculosis* [55] but long-read sequencing could provide a more comprehensive understanding of these regions, their contribution to the resistance phenotype, and the pathogenesis of the strain [52]. However, until very recently (after this project was initiated) the accuracy of LR nanopore sequencing was limited because of error-prone homopolymer regions [56]. The R.10 flow cells, designed to provide optimal translocation speed for homopolymer sequences within pores, and the improvements in basecallers (namely Guppy) are improving the sequence accuracy, thereby the variant calling and subsequent drug-resistance prediction [57].

The bioinformatics analysis for anti-TB drug resistance prediction and strain typing for molecular epidemiology of MTB, particularly for LR sequence data, remains challenging. There must be a balance between finding as many variants as possible, even if in minor populations, and correctly predicting the susceptibility patterns. Additionally, differences in variant databases can impact the predicted resistance profiles while using different tools. These variants should be further evaluated *in vitro* (e.g. determine the need for compensatory variants to express the resistant profile). For LR, the accuracy of the results will potentially be improved with improved chemistry and bioinformatics (e.g. R10 flowcells, recent versions of Guppy). While WGS has been used as a complementary diagnostic method guiding phenotypic testing, the implementation of WGS-diagnosis needs further standardization and extensive validation studies for the currently in use bioinformatics tools for MTB. Nevertheless, we could show that polishing LR assemblies improved the genome quality and that reproducible phylogeny can be achieved using cgSNP approaches, especially for LR sequence data.

## Supplementary Data

Supplementary material 1
